# Central retinal vein occlusion concomitant with dengue fever

**DOI:** 10.1186/s40942-016-0027-x

**Published:** 2016-01-20

**Authors:** Punithamalar Velaitham, Nandini Vijayasingham

**Affiliations:** Ophthalmology Department, Hospital Tuanku Ja’afar Seremban, Jalan Rasah, 70300 Seremban, Negeri Sembilan Malaysia

**Keywords:** Central retinal vein occulsion, Dengue fever, Ophthalmic complications of dengue fever

## Abstract

**Background:**

Dengue virus infection is on the rise and there is increasing number of ocular complications that are being reported. Most common ocular complications are macular edema, macular hemorrhages, and foveolitis. There are case reports on branch retinal vessel occlusions. Most of the ocular complications are attributed to the bleeding tendency and transudative process in dengue viral infection. This is a case report of ischemic central retinal vein occlusion (CRVO) concomitant with dengue fever.

**Case presentation:**

A 41 year old Malay female was admitted to medical ward and diagnosed to have “dengue fever with warning signs”. On the day of admission she noted sudden onset of right eye blurring of vision. She presented to our clinic 1 week later. Ocular examination revealed right eye visual acuity of <20/1000 and ischaemic CRVO with macular edema. She had no other risk factors to develop retinal vein occlusion. She progressively developed proliferative retinopathy and received multiple laser therapy. There was no anterior segment neovascularization. However, her vision improved to only 20/400 despite of resolution of macular edema and new vessels elsewhere.

**Conclusion:**

Dengue virus infection is known to cause thrombocytopenia which can result in hemorrhagic events. It can also cause procoagulant state which can result in thrombotic events secondary to immune reaction. Awareness among treating physicians of such ocular complication which can result in significant morbidity for patient is necessary.

## Background

Dengue is one of the most important arthropod-borne viral diseases in terms of human morbidity and mortality [1]. The number of reported dengue viral infections in Malaysia is alarming and on the rise [2]. Pathogenesis of severe dengue illness is attributed to increased vascular permeability and excessive bleeding caused by thrombocytopenia. There is an increasing evidence suggesting that abnormalities in the coagulation and fibrinolytic systems are also involved in dengue pathogenesis. Ocular complications following dengue fever include retinal hemorrhage, macular edema, retinal vasculitis and uveitis [3]. We report a case of central retinal vein occlusion (CRVO) in dengue fever.

## Case report

A 41 year old Malay female with no prior medical illness, was admitted to medical ward with the complain of fever, headache and reducing platelet and increasing hematocrit for 3 days duration. Her Dengue NS-1 antigen was reactive which confirms dengue virus infection. She was diagnosed to have ‘dengue fever with warning signs’ based on WHO classification of levels of severity [1]. On admission she complained of right eye blurring of vision for 1 day. Fundus examination was done by medical officer in Medical ward and was documented normal. Therefore she was not referred to Ophthalmology department during the hospital stay. She was treated with fluid resuscitation and was discharged after 5 days. However, right eye blurring of vision persisted and then she was referred to the ophthalmologist. She presented to our eye clinic with right eye blurring of vision for 8 days. It was sudden in onset and non-progressive.

**Fig. 1:**
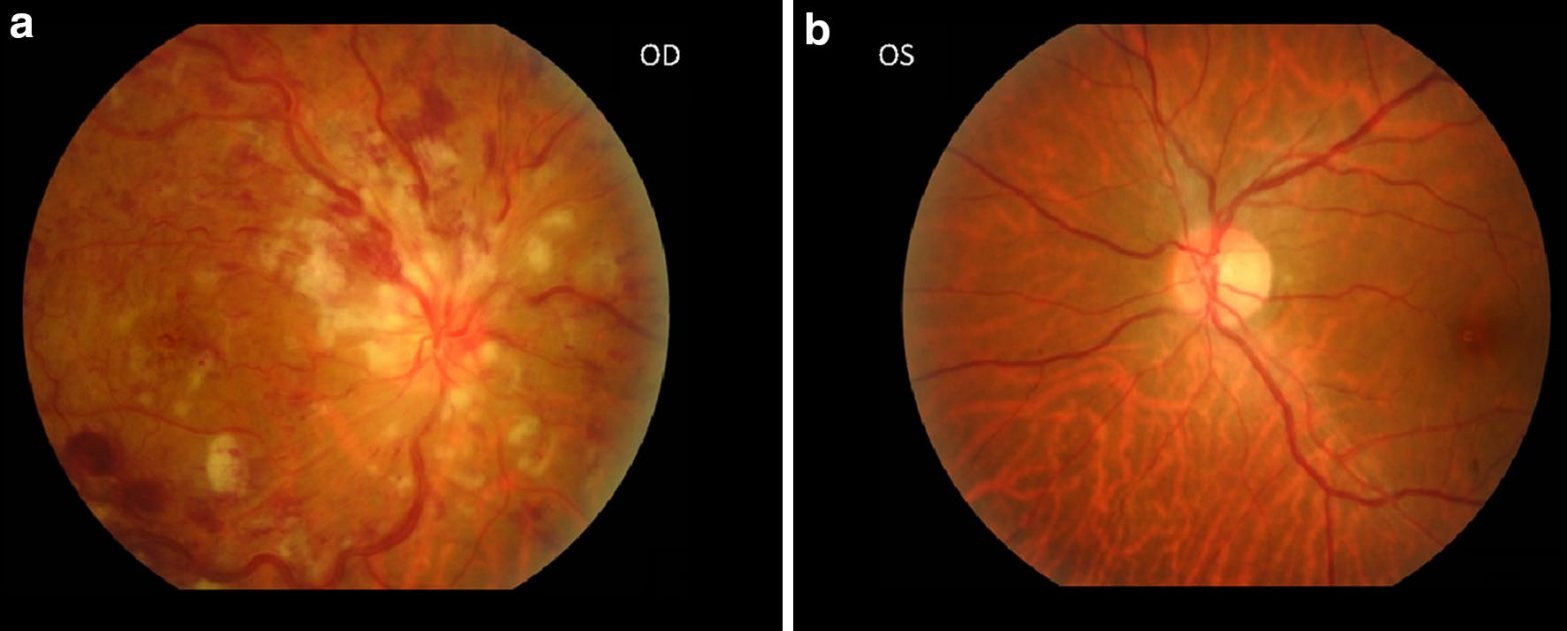
Optic disc centred both eye fundus pictures. **a** Right eye—note the dilated tortuous veins, retinal hemorrhages, optic disc edema, and cotton wool spots around the disc. **b** Left eye—normal fundus

On examination, patient was alert, comfortable and vital signs were stable. Ocular examination noted best corrected visual acuity in right eye was <20/1000 and left eye was 20/25. Intraocular pressure was normal in both eyes. Relative pupillary afferent defect was detected in the right eye. Right eye fundus showed typical features of CRVO—dilated, tortuous retinal veins and retinal hemorrhages in all four quadrants, optic disc swelling, cotton wool spots and macular edema. Left eye examination was normal (Fig. 1).

**Fig. 2:**
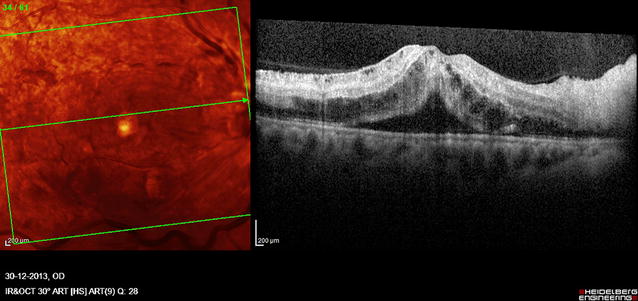
OCT of right eye macula. Note the retinal thickening, cystoid spaces within retina, and subretinal fluid

Optical coherence tomography (OCT) of right eye macula disclosed cystoid spaces with edema, retinal thickening and subretinal fluid (Fig. 2).

Further assessment to identify the risk factors for CRVO were done. She was not on any hormonal contraception. Blood investigations were done namely fasting serum lipid, fasting blood sugar, renal profile, full blood count (post recovery), anti-cardiolipin antibodies (ACA), Anti-Beta-2-Glycoprotein 1 (2GP1), Protein C activity, Protein S activity, Antithrombin activity, Activated Protein C Pathway which were normal. Serum electrophoresis shows no paraprotein band and no immune paresis. Her full blood count (FBC) trend during hospital stay showed increasing hematocrit and reducing platelet which peaked on day 6 of illness and then gradually improved (Table 1). Her symptoms onset was during early period of the illness when her hematocrit and platelet levels were still acceptable.

**Table 1:**
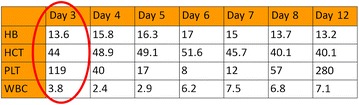
Full blood count results during hospital stay

The result on day 3 during which the symptoms started. It occured early in the disease onset. There is progressive reduction in hemotocrit and platelet level with peak on day 6 of illness which later gradully improves

*HB* hemoglobin, *HCT* hematocrit, *PLT* platelet, *WBC* white blood cell

**Fig. 3:**
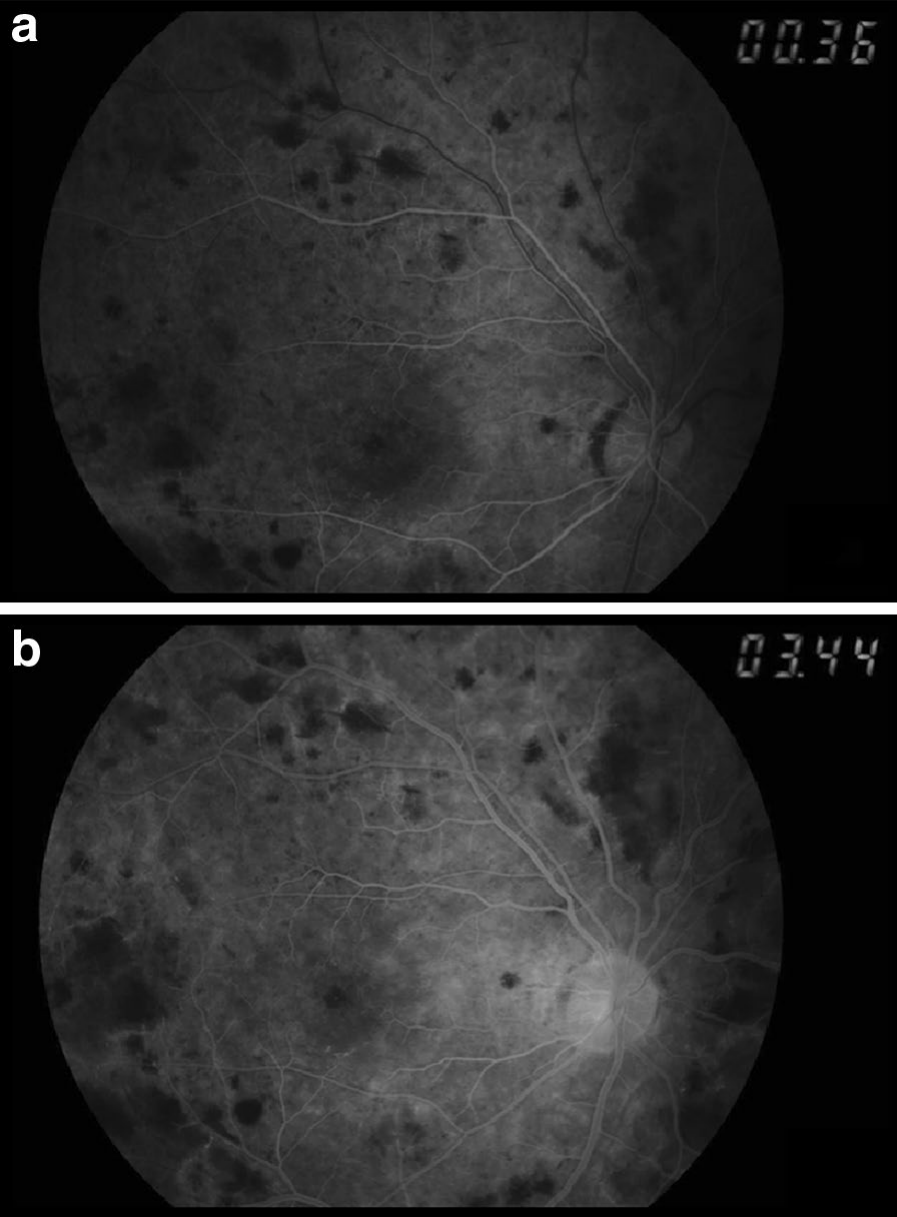
Fundus fluorescein angiography of right eye **a** shows delayed venous filling, **b** less than ten disc diameters of capillary dropout and masking

Six weeks later, fundus fluorescein angiography was done and there was delayed venous filling, less than ten disc diameter area of capillary dropout and areas of masking by retinal hemorrhages (Fig 3). Patient was planned for repeat FFA, however her right eye progress to develop proliferative retinopathy—new vessels elsewhere on the posterior pole which was treated with panretinal photocoagulation. There was no new vessels on the iris or angle noted. The macular edema improved drastically and was monitored with OCT imaging. Unfortunately, her vision did not improve. Her best corrected visual acuity of right eye is 20/400 and left eye 20/25.

## Discussion

Ophthalmic complications following dengue fever are not uncommon. The spectrum of ophthalmic manifestations would lead one to conclude that several pathophysiologic processes are involved [3], namely thrombocytopenia, increased vascular permeability and inflammation. These changes manifest as anterior uveitis, retinal hemorrhage [4, 5], periphlebitis and macular edema [6, 7].

In this case, the thrombotic event may be related to immune response and down regulation of antithrombin. There are two different mechanisms described for this. *Cabello* et al. described that there is downregulation of cytoprotective protein C pathway in human endothelial vascular cells in Dengue infection. Profound endothelial damage caused by anti–NS1 antibody cross reaction with vascular endothelium result in reduction of thrombomodulin on endothelial surface [8]. This in turn reduces activated protein C (APC) generation. Downregulation of APC anticoagulant pathway promotes thrombosis [8]. Conversely, *Mairuhu* et al. measured plasma level of plasminogen activator inhibitor type 1 (PAI-1) concentrations in patients who were admitted with clinically suspected severe dengue virus infection. They demonstrated an increase in PAI-1 plasma level in individuals with dengue virus infection and in particular in those with poor clinical outcome. Primary role of PAI-1 is to inhibit fibrinolytic activity in vivo [9]. These factors attribute to the procoagulant state and are associated with greater risk for thrombosis [8].

Similarly, several major vessel thrombotic events were reported during dengue infection in Brazil. These patients account for 5.4 % of all dengue inpatients. Severe dehydration, a well known condition associated with thrombotic events, was not detected in any of these patients. Likewise, thrombotic events in these patients were thought to be due to loss of endothelial non-thrombogenic protective factors early in the course of the disease [10].

In this case the onset of symptoms did not coincide with highest hemoconcentration state or lowest platelet level. It occurred early in the disease process which would suggest that immune reaction could be the cause of thrombosis. The most common ocular manifestation of dengue virus infection is macular edema, macular hemorrhage and foveolitis [11]. Retinal hemorrhages are common and can be associated with cotton wool spots. In this patient, cotton wool spots were numerous and around peridisc region. This is in keeping with CRVO and reflects the severity of retinal ischemia. As a consequences this patient developed proliferative retinopathy which is seen in 9 % of CRVO patients within 6 months from onset [12]. Early commencement of laser therapy could have reduced vascular endothelial growth factor level in the vitreous and prevented anterior segment neovascularization.

This patient was treated with panretinal photocoagulation for proliferative retinopathy. Intravitreal anti-vascular endothelial growth factor (anti-VEGF) injection is the preferred modality of treatment in some countries [13]. However, in our setting it is yet to be approved for the use in macular edema secondary to CRVO. The macular edema improved drastically within 2 months which was confirmed with OCT imaging and her vision improved from <20/1000 to 20/400.

## Conclusion

To our best knowledge, this is the first case report of ischemic CRVO in Dengue fever. This adds to the evidence that Dengue fever has an immunologic component in its pathogenesis. Future study is warranted to ascertain if immunosuppressive therapy is beneficial in such patients. Attending doctors should be aware of diverse ophthalmic complications of dengue fever and co-manage with ophthalmologist to prevent and reduce morbidity.

## Consent

Written informed consent was obtained from the patient for publication of this Case report and any accompanying images.
